# Supplementary material for: Chromosome-Level Genome of Simulium vittatum Links Black Fly Cytogenetics to Genome Organization and Evolution
Source: bioRxiv. 2026 Sep 21:2026.09.08.750124. Preprint. [Version 2] doi: 10.64898/2026.09.08.750124 (PMC13622007; doi:10.64898/2026.09.08.750124)
Supplement: Supplementary file 2 [file media-2.pdf]

Suppl. Table S2: Distribution of long-read sequencing lengths for the Simulium vittatum genome, summarized in 1 kb bins.

Read length interval (bp)	Read count	Percentage of reads (%)
0-999	778008	10.57
1000-1999	543619	7.39
2000-2999	809356	11.00
3000-3999	726413	9.87
4000-4999	542887	7.38
5000-5999	408802	5.55
6000-6999	309481	4.20
7000-7999	247144	3.36
8000-8999	204668	2.78
9000-9999	174202	2.37
10000-10999	151672	2.06
11000-11999	136091	1.85
12000-12999	123808	1.68
13000-13999	114877	1.56
14000-14999	107658	1.46
15000-15999	104913	1.43
16000-16999	103718	1.41
17000-17999	106217	1.44
18000-18999	111986	1.52
19000-19999	117739	1.60
20000-20999	121175	1.65
21000-21999	122370	1.66
22000-22999	119293	1.62
23000-23999	112876	1.53
24000-24999	104850	1.42
25000-25999	94886	1.29
26000-26999	85308	1.16
27000-27999	76871	1.04
28000-28999	68870	0.94
29000-29999	61062	0.83
30000-30999	55039	0.75
31000-31999	48888	0.66
32000-32999	44033	0.60
33000-33999	39628	0.54
34000-34999	35733	0.49
35000-35999	31350	0.43
36000-36999	28110	0.38
37000-37999	24982	0.34
38000-38999	21155	0.29
39000-39999	17904	0.24
40000-40999	14321	0.19
41000-41999	11423	0.16
42000-42999	9016	0.12

Read length interval (bp)	Read count	Percentage of reads (%)
43000-43999	7632	0.10
44000-44999	6901	0.09
45000-45999	6273	0.09
46000-46999	5830	0.08
47000-47999	5247	0.07
48000-48999	4985	0.07
49000-49999	4579	0.06
50000-50999	4317	0.06
51000-51999	3830	0.05
52000-52999	3645	0.05
53000-53999	3379	0.05
54000-54999	3063	0.04
55000-55999	2810	0.04
56000-56999	2505	0.03
57000-57999	2390	0.03
58000-58999	2176	0.03
59000-59999	2015	0.03
60000-60999	1737	0.02
61000-61999	1543	0.02
62000-62999	1407	0.02
63000-63999	1236	0.02
64000-64999	1175	0.02
65000-65999	1031	0.01
66000-66999	904	0.01
67000-67999	764	0.01
68000-68999	667	0.01
69000-69999	609	0.01
70000-70999	535	0.01
71000-71999	495	0.01
72000-72999	400	0.01
73000-73999	397	0.01
74000-74999	305	0.00
75000-75999	262	0.00
76000-76999	239	0.00
77000-77999	246	0.00
78000-78999	178	0.00
79000-79999	224	0.00
80000-80999	165	0.00
81000-81999	152	0.00
82000-82999	122	0.00
83000-83999	127	0.00
84000-84999	84	0.00
85000-85999	96	0.00
86000-86999	85	0.00
87000-87999	65	0.00
88000-88999	59	0.00

Read length interval (bp)	Read count	Percentage of reads (%)
89000-89999	60	0.00
90000-90999	66	0.00
91000-91999	68	0.00
92000-92999	48	0.00
93000-93999	52	0.00
94000-94999	42	0.00
95000-95999	34	0.00
96000-96999	29	0.00
97000-97999	36	0.00
98000-98999	23	0.00
99000-99999	25	0.00
1e+05-100999	27	0.00
101000-101999	30	0.00
102000-102999	19	0.00
103000-103999	20	0.00
104000-104999	16	0.00
105000-105999	20	0.00
106000-106999	8	0.00
107000-107999	16	0.00
108000-108999	9	0.00
109000-109999	14	0.00
110000-110999	12	0.00
111000-111999	11	0.00
112000-112999	7	0.00
113000-113999	8	0.00
114000-114999	4	0.00
115000-115999	10	0.00
116000-116999	6	0.00
117000-117999	5	0.00
118000-118999	4	0.00
119000-119999	3	0.00
120000-120999	5	0.00
121000-121999	14	0.00
122000-122999	12	0.00
123000-123999	3	0.00
124000-124999	4	0.00
125000-125999	1	0.00
126000-126999	6	0.00
127000-127999	4	0.00
128000-128999	6	0.00
129000-129999	2	0.00
130000-130999	4	0.00
131000-131999	3	0.00
132000-132999	4	0.00
133000-133999	2	0.00
134000-134999	4	0.00

Read length interval (bp)	Read count	Percentage of reads (%)
135000-135999	3	0.00
136000-136999	1	0.00
137000-137999	1	0.00
140000-140999	3	0.00
141000-141999	1	0.00
143000-143999	1	0.00
144000-144999	2	0.00
145000-145999	1	0.00
146000-146999	1	0.00
148000-148999	1	0.00
151000-151999	3	0.00
156000-156999	1	0.00
161000-161999	1	0.00
163000-163999	1	0.00
180000-180999	1	0.00
183000-183999	1	0.00
184000-184999	1	0.00
197000-197999	1	0.00
223000-223999	1	0.00
271000-271999	1	0.00
329000-329999	1	0.00
